# The impact of a telehealth platform on ABA-based parent training targeting social communication in children with autism spectrum disorder

**DOI:** 10.1007/s10882-022-09839-8

**Published:** 2022-03-29

**Authors:** Jenny Ferguson, Katerina Dounavi, Emma A. Craig

**Affiliations:** School of Social Sciences, Education & Social Work, Queen’s University of Belfast, 69-71 University Street, BT7 1HL Belfast, Northern Ireland

**Keywords:** Telehealth, Applied behaviour analysis, Autism spectrum disorder, Parent training, Communication

## Abstract

Interventions based upon applied behaviour analysis (ABA) have been shown to be best practice for children with autism spectrum disorder. However, in many parts of the world there is a shortage of appropriately trained behaviour analysts. Telehealth is a potential solution to increasing access to ABA. Our study assessed the use of telehealth to provide parent training in naturalistic teaching strategies designed to increase child communication skills. Five parent child dyads took part in the training, utilising didactic training and synchronous coaching. Parents could be trained to a high level of fidelity and viewed the training favourably. Children showed variable gains in communication and improved positive affect. The project was cost effective in comparison with traditional training models.

One ABA-based method of increasing skills in social communication (e.g., verbal communication, joint attention or imitation) is naturalistic teaching (Hume et al., 2021a, 2021b; Ontario Association of Behavior Analysts, 2017; Wong et al., 2014). Naturalistic teaching embeds teaching into everyday activities (e.g., in play, art activities or during mealtimes), allowing behaviour to be controlled by a variety of natural consequences and prioritisation of child interests. Naturalistic teaching enables adults to bring about and reinforce targeted behaviour under relevant motivating operations. Additionally, naturalistic teaching has been shown to facilitate generalisation and maintenance of skills (Charlop-Christy & Carpenter, 2000).

Teaching behaviour which remains under the control of natural contingencies of reinforcement should be the primary goal of behavioural intervention (Leaf et al., 2016) and having a teaching system which promotes this can ensure meaningful behaviour change. One early social communication skill targeted in naturalistic interventions is requesting preferred items or activities (from here on referred to as “manding” or “mands” as per Skinner’s taxonomy of verbal behaviour; Skinner, 1957). Enabling children with ASD to communicate their wants and needs can accelerate the learning of subsequent verbal communication and limit behaviour that challenges which can be learnt in lieu of an appropriate communication system (DeSouza et al., 2017; Howell et al., 2019; Ward & Shukla Mehta, 2019). Additionally, eye gaze as a form of a mand holds a prominent role in early learning of effective communication in natural settings. Atypical development in this area becomes evident very early in life (Tanner & Dounavi, 2021) and may have a detrimental effect on the amount and quality of interactions across development (Krstovska-Guerrero & Jones, 2016). This is because eye contact, often called eye gaze, is considered a behaviour cusp which can open the door to other socio-communicative behaviours and has therefore been targeted prior to teaching vocal or alternative manding targets (Tanner & Dounavi, 2020).

One common priority in naturalistic teaching is the inclusion of parent and caregiver training in intervention (Akamoglu & Meadan, 2018; LeBlanc et al., 2006; Schreibman et al., 2015). When paired with parent training, naturalistic teaching can further allow for socially validated behaviour change to occur in ecologically relevant environments (Meadan & Daczewitz, 2015; Vismara et al., 2013) and, as parents form a large part in the natural contingencies of reinforcement which exist in a child’s world, including parents in intervention can assist in the generalisation of behaviour change (Lang et al., 2009). Unfortunately, global shortages of appropriately trained professionals, (e.g., Board Certified Behaviour Analysts or BCBA® educated at master’s or doctoral level) have detrimental effects on accessibility of such training in many countries (Keenan et al., 2015). Whilst the BCBA® is a general credential in behaviour analysis and does not specify expertise in ASD intervention, many BCBA® practitioners have specialised in this area and may be best placed to provide training in naturalistic behavioural strategies (Behavior Analyst Certification Board, 2020). Shortages in BCBAs® are evident across much of the world including in the UK and Ireland. For example, as of February 2022, in Scotland there were only six BCBA®/BCBA-D®s, in Northern Ireland there were 34 and in the Republic of Ireland there were 195 (Behavior Analyst Certification Board, 2022). The Behaviour Analyst Certification Board’s decision to discontinue international certification from 2023 (Behavior Analyst Certification Board, 2021) may decrease the number of professionals with a BCBA credential in areas outside the US and Canada. Shortfalls in BCBA® numbers are also magnified in remote areas of well served countries, for example in Ontario, Canada, as of February 2022 there is a total of 1,272 BCBAs® or BCBA-Ds® of which more than half reside in either the Greater Toronto Area or Ottawa, leaving a large rural area underserved (Behavior Analyst Certification Board, 2022). The repercussions of such shortages may be felt in long wait lists for “early” services, or worse still, children may simply not be able to access vital support in certain geographical regions (Keenan et al., 2015; Salomone et al., 2016).

It is unquestionable that possible solutions need to be explored in order to expand the reach of professionals and attempt to close the gap between service need and delivery capacity. Developing a model that allows professionals to practise outside traditional face-to-face formats will increase capacity and allow provisions to be delivered in previously un-serviceable regions. One promising delivery model is telehealth. Telehealth is the use of communication technology to provide training and treatment for health-related conditions (World Health Organization, 2021). Researchers in ABA have recognised the potential of this delivery platform and have assessed commonly used ABA-based strategies delivered via a telehealth platform (see Ferguson, Craig, & Dounavi, 2019 for full review), including strategies designed to increase social communication and parent training packages (e.g., Ingersoll et al., 2017; Ingersoll et al., 2016; Vismara et al., 2009).

Telehealth-based studies have been conducted which targeted both parental fidelity in completing trained strategies and child social communication targets including requesting, labelling, social referencing and joint attention (e.g., Meadan & Daczewitz, 2015; Vismara et al., 2013, 2016). Results from this body of evidence are mixed. High levels of trainee fidelity have been be achieved (Ferguson et al., 2019; Neely et al., 2017) and research adopting a group design has had promising outcomes (Ingersoll & Berger, 2015; Ingersoll et al., 2016; Vismara et al., 2016). Despite this, on an individual level, child outcomes are varied. Ferguson et al. (2019) found only 54% of studies reported favourable outcomes for all participants and Neely et al. (2021) stated 45% of single subject research focusing on communication interventions demonstrated non-effects for child outcomes. Whether this limitation in outcomes is a result of heterogeneity common to a naturalistic teaching model or due to specific difficulties in translating effective naturalistic teaching practices to a telehealth platform (i.e., not being able to readily model behaviour or control the many competing variables which may exist in less structured environments) are important questions to ask. The completion of additional high rigor research adopting a single subject design is consequently warranted. Such research will provide information on individual differences in behaviour change which can be masked by the overall improved performance of the group (Dounavi & Dillenburger, 2013).

Despite the suitability of a telehealth platform to provide training to remote locations globally, research on its efficacy is somewhat limited. International replications are important additions to the literature as they provide an indication of the success of the telehealth platform under circumstances where their utility will be most beneficial. Previous international studies have focused on disseminating US-based expertise (e.g., Barkaia et al., 2017; Neely et al., 2020; Tsami et al., 2019). Most recently, Neely et al. (2020) conducted a two-tier pyramidal training in Japan. The researchers taught a doctoral student “coach” naturalistic teaching strategies designed to increase communication. This coach subsequently taught three other interventionists how to implement the strategies. The authors found that all interventionists were successfully trained to a high level of fidelity but child social communication targets (requesting) varied, mirroring US-based findings.

Whilst these US-based international studies are vital and welcomed additions to the literature, there is a dearth of research being produced by global researchers (i.e., studies in which neither researcher nor participants are located within the US). This has been highlighted in a recent review (Sivaraman & Fahmie, 2020). Emerging research, although limited, has indicted international replications are possible (e.g., Craig et al. 2021) but to date and to the best of our knowledge there have been no studies published by researchers outside of the US investigating solely a telehealth platform to provide training to parents in naturalistic interventions. Expanding the reach of professionals who are located in the same or neighbouring countries as the trainees could have a number of unique benefits. For example, this will allow synchronous training to be conducted in countries which are not in convenient time zones for researchers from the US. Furthermore, it will enable professionals to have a unique understanding of cultural considerations in the countries serviced and country specific knowledge on issues relating to child welfare and safeguarding. The production of country specific cost-effective research demonstrating the success of telehealth may also have more traction in encouraging funding of ABA services in regions where this is not currently commonplace.

In addition to this, our study uniquely includes measures of child affect. Child affect/indicators of mood have become more common in ABA literature in recent years (see Ramey, et al. 2019 for review) for constituting a valuable indication of the effectiveness of interventions at improving quality of life for individual with ASD. These indicators can be operationally defined and serve to measure child enjoyment during sessions (i.e., an instance of facial expression usually associated with happiness, smile, laugh etc.; Greiger et al. 2012). To date, none of the published research using telehealth has included affect as a dependent variable. This inclusion may provide an important measurement of child social validity missing from the previous research base.

In our study we primarily aimed to fill gaps in current knowledge by assessing a naturalistic training package delivered via telehealth in an international setting. Data were scored solely from videos sent in by parents and recorded independently from the coaching sessions with the trainer not present, a rare approach in existing research, which provided measures of parental generalisation of strategies in the absence of the trainer or feedback. Parent’s use of teaching strategies designed to increase communication in play sessions was assessed for fidelity and used as the primary dependent variable for the study. These data were used to answer the main research question: “Can a telehealth model be used to train parents to use ABA-based naturalistic teaching strategies to a high level of fidelity?”. Further parent measures of social validity were collected which aimed to determine: “Do parents rate the training favourable?”. As a secondary aim, we assessed child outcomes which included increases in child social communication behaviour, specifically mands using eye gaze with a parent and the use of individualised communication targets, alongside a measure of child affect. These data were assessed to answer the following two research questions: “Does this training result in increased communication skills for the children?” and “Does this training result in increased levels of positive affect for the children?”. Finally, a cost analysis was completed to answer the question: “Is a telehealth platform cost-effective when compared to a face-to-face training model?”.

## Method

### Participants and Settings

Participants were recruited via a poster advertisement which was shared using the following channels: (a) an online advertisement on social media platforms (e.g., The Centre of Behaviour Analysis at Queen’s University Belfast Facebook page and parental support Facebook pages ABA4ALL or ABAIreland), (b) through professional contacts, and (c) through email lists of organisations providing parental support.

Parents were recruited for the study if they met the following criteria: (a) were the primary caregiver of an individual with a diagnosis of ASD, (b) had access to a reliable internet connection capable of conducting video-calls, (c) were willing to commit to at least 2 h per week of training and additional time to practise and provide consent to do so, and (d) had not taken any official prior training in behaviour analytical principles. If unofficial training had been undertaken (e.g., courses not accredited or verified by Applied Behaviour Analysis International), such as a short online course, parental knowledge and practical skills were assessed to determine whether parents were eligible. Such parents were asked to complete the multiple-choice pre-test (described in subsequent sections). If results of the test were greater or equal to 80%, parents would be asked to submit a tester video (following the same procedures outlined in the baseline videos described in the subsequent section). This 80% cut-off in tests of knowledge was deemed feasible and was decided by examining findings from past telehealth research adopting a didactic training (e.g., see Hamad et al., 2010). The submitted video demonstrated interactions with their child, which would be scored for each of the strategies taught. Videos were scored for each of the strategies included in study, parents had to score less than 80% to progress with the project. Parent demographic information is summarised in Table 1.

**Table 1:** Demographic details of parent participants

Parent	Age	Sex	Education Level	Occupation
Elaine	34	Female	University Degree	Career break, clerical work for University
Christina	33	Female	PhD	University lecturer
Jill	39	Female	Undergraduate degree. Master’s degree in Education 2/3rds complete but on break.	Career break from secondary school teacher
Diane	37	Female	College Diploma	Stay at home Mum
Sarah	44	Female	Undergraduate degree	Stay at home Mum

In order to be eligible, child participants had to: (a) have a diagnosis of ASD as documented from an official diagnostic tool, such as The Autism Diagnostic Observation Schedule-2 (ADOS-2) (Lord et al., 2012) or The Autism Diagnostic Interview-Revised (ADI-R) (Rutter et al., 2003), (b) be younger than 84 months old (7 years) at the time of recruitment, (c) have considerable difficulties in communication and social interaction identified through a pre-study behavioural assessment (i.e., The Verbal Behavior Milestones Assessment and Placement Program; VB-MAPP; Sundberg, 2008). The VB-MAPP is an assessment measuring child verbal behaviour against age rated developmental milestones, which additionally includes a Barriers Assessment to determine the impact challenging behaviour may have. Prior to starting the project assessments based upon the VB-MAPP were conducted with every participant. Child demographic information is displayed in Table 2. A total of nine interested families were excluded during a pre-screening stage. This was due to the following reasons: too much experience in ABA (3 families) (e.g., completion or undertaking an MSc), no ASD diagnosis (2 families), child was too old at intake (3 families), parent wanting to focus on skills not included in the project (1 family). A total of seven parent/child dyads were recruited for the research. One family did not submit the required baseline videos and was therefore not counted in attrition figures. One further parent withdrew from the study after completing didactic training due to work commitments and personal circumstances.

**Table 2:** Demographics and individualised targets for child participants

Name	Age	Sex	Diagnosis and Tools	Current Services	Communication target
Patrick	4years 5months	Male	ASD and Global Developmental Delay ADOS-2	Specialist nursery SLT	One-word vocal requests
Kostas	3years 6months	Male	ASD, Speech and Language Impairment ADI-R	Mainstream nursery SLT and OT	Two-three-word vocal requests
Leanne	3years 3months	Female	ASD ADOS-2	Home tuition over summer months	One-word vocal requests
Sean	2 years 11 months	Male	ASD ADOS-2	Specialist nursery	One-word signs
Eamonn	6 years 1 10 months	Male	ASD DSM-V	Mainstream school, home tuition	Two-three-word vocal requests

In order to maintain anonymity all parents and children have been provided with a pseudonym. Elaine was 34 years old from rural Northern Ireland. Her son Patrick was 4 years 5 months. Patrick’s scores on the VB-MAPP indicated that he had a very limited mand repertoire, scoring 1 out of 5 in 0-18-month level 1 mand section. Patrick did not consistently request preferred items but would lead his mum’s hand to indicate what he wanted or would retrieve the item himself. Patrick could produce speech sounds and several whole word approximations, although these were not yet under echoic control and occurrences of these were rare. He demonstrated limited spontaneous eye gaze in both the assessment and baseline sessions.

Christina was Southern European but resided in Scotland; Christina’s husband, Demetri, was involved in the project by taking part in all didactic sessions. Their son Kostas was 3 years and 6 months; Kostas currently communicated primarily using one-word utterances. He met full criteria for all 5 milestones on level 1 of the VB-MAPP and 4 out of 5 criteria on level 2. He was able to echo multisyllabic utterances and had some limited ability to request using more than one word and for others to use actions but did not meet the criteria for this milestone. He would establish eye gaze with an adult when prompted but spontaneous occurrences of this were rare in the assessment and baseline sessions. Kostas displayed some non-engagement with the demands presented during the VB-MAPP assessment, which acted as a barrier to a full developmental picture being obtained.

Jill was 39 years old and lived in rural Republic of Ireland. Her daughter Leanne was 3 years 3 months old. Leanne had a limited mand repertoire and only demonstrated two vocal mands during the assessment and baseline videos; these were “mama” and “help” when presented with a non-preferred task on two occasions during a baseline video. As such she scored 1 out of 5 in 0-18-month level 1 mand section Leanne did not demonstrate the ability to echo words but did vocalise speech sounds more than 25 times per hour, she could emit some whole word approximations and demonstrated limited eye gaze.

Diane was 37 years old from rural Northern Ireland. Diane’s son Sean was 2 years and 11 months old. Sean had a very limited verbal repertoire, he produced very few speech sounds and no whole word approximations. None of his sounds were under echoic control. In the VB-MAPP assessment Sean scored 0 out of 5 in 0-18-month level 1 mand section and did not have a system of communication in place. Instead, Sean would communicate what he wanted either by leading his mum to the item, giving the item to her or on occasion would engage in behaviour considered challenging, such as crying, screaming and flopping to the ground.

Sarah was 44 years old and from rural Republic of Ireland. Her son Eamonn was 6 years and 10 months old upon recruitment. Eamonn was able to communicate using one-word or short sentences including requesting some actions such as “tickle me” but independent occurrences of a variety of responses of this nature were relatively low at baseline and there was some evidence of prompt dependency. Eamonn met full criteria for all 5 milestones on level 1 of the VB-MAPP and 4 out of 5 criteria on level 2 and demonstrated a good echoic repertoire including multisyllabic utterances. He scored highly on the Barriers Assessment component of the VB-MAPPS, especially around escape/avoidance of demand. This was evident during the second assessment meeting and may have limited the ability of this assessment to provide a full developmental picture.

Parents took part in all training sessions in the home environment, during didactic sessions only the parent(s) and the trainer were present. The children joined the sessions during the coaching stage. Prior to the commencement of this stage, parents and the trainer discussed appropriate areas for the coaching sessions to be conducted based upon the child’s preferences. All sessions took the form of a “play session” and utilised toys and activities already present in the home environment. Areas had to have access to the internet and often a pre-identified location for the placement of the video camera or phone was agreed. The overarching principle of the training was to focus on the motivation of the child. This meant that parents were trained to generalise skills by identifying and utilising motivation of the child in several locations around their house.

### Training and Materials

Parents used their personal computers, laptops and tablets during the study. If families owned a webcam or device capable to filming, these were checked for resolution and used if acceptable. If they did not own a web-cam or video recording device, they were provided with a Logitech C920 HD Pro Webcam-Full HD 1080p prior to the study. Bluetooth headphones with inbuilt microphone were provided to each family on order for live feedback to be provided during the coaching stage of the study (JBL T110BT In-Ear Wireless Headphones).

All training sessions were completed by the first author, who had an undergraduate degree in Psychology, a post graduate teaching qualification and a master’s degree in ABA. She was a Board Certified Behaviour Analyst (BCBA®) with over 10 years’ experience delivering behaviour analytic interventions and training parents and professionals. She was currently perusing a research PhD. All training documents were written by the first author under the oversight of the second author who was a BCBA-D® with nearly 20 years’ experience in ABA and ASD and over 10 years in telehealth. Whilst conducting the training sessions, the trainer was located in a University or home office in Belfast, Northern Ireland. Parents were located on average 118 miles away from the trainer (Range: 30–228 miles). The trainer used a Lenovo ideapad 330 laptop and built-in webcam for all sessions. All didactic training and live feedback sessions were conducted via Skype™ video conferencing software and in line with the General Data Protection Regulation (GDPR). Materials were stored and accessed by parents via the online learning platform Canvas. The Canvas platform was able to host all the training materials which were included for each didactic training session: a PDF, a Prezi presentation and two quizzes. Videos recorded by parents were uploaded to secure servers Dropbox™ or WeTransfer, both GDPR compliant encrypted file sharing sites. Additional communication with parents was conducted via email with the trainer utilising Office 365. An overview of the software used can be found in the supplementary materials (Supplementary Table 1).

### Research Design

 The study adopted a concurrent multiple probe design across participants. Multiple probe designs forgo the requirement for repeated measures during baseline, instead behaviours were “probed” to determine a lack of behaviour change in subsequent participants’ baselines. This design was chosen based on feasibility factors (i.e., it was deemed reasonable to ask parents to provide three to four repeated baseline videos prior to starting the study). Following the recommendations of Carr (2005), data have been graphed concurrently. That is data taken from videos submitted by parents have been graphed in temporal order so that relative relationships of data can be determined in relation to other participants in the study and in relation to the commencement of each training strategy used in the project and changes observed in dependent variables.

### Pre-intervention Assessments and Baselines

Prior to commencing the intervention, the trainer and the parent met via Skype™ on one or two occasions. Each meeting lasted approximately 1 h and aimed to determine the child’s current level of functioning based upon communication sections of the VB-MAPP (Sundberg, 2008). Scores were calculated from direct observations of the baseline videos, live testing during the meetings via video-conferencing and through discussions with parents. In order to complete the assessment, parents were provided with a written overview and instructions. They were then coached live on how to test each skill. Additionally, the VB-MAPP Barriers assessment was discussed with each parent. The results of these assessments and the baseline videos were used to determine individualised communication targets for each participant, rather than provide an extensive assessment.

The first two baseline probes for each participant were collected concurrently across a period of 2 weeks. Written instructions were provided which asked parents to send a 12-15-minute video depicting them playing and communicating with their child in their usual way and no feedback was provided. Prior to starting the training, and after parents in the prior tier of the study demonstrated an increased fidelity in any of the strategies taught, parents were asked to send the final baseline video. They subsequently completed a multiple-choice test to assess their knowledge of ABA principles and theory. This test was also used to determine the eligibility of two parents who had completed some prior training in ABA. The test consisted of 20 multiple-choice questions delivered via the Canvas platform. Parents completed the test independently and were not given any specific feedback apart from their overall score (test questions can found in supplementary materials Table 2).

### Dependent Variables, Data Collection and Analysis

Independently recorded videos sent by parents provided the main source from which data were analysed for all parent and child dependent variables. Each video was standardised to disregard the first 2 min for reactivity and the remaining 10 min were scored. Instructions provided during baseline informed parents how to set up the videos to gain the best views. Parents were also allowed to ask an extra person to film the sessions as this could provide a clearer picture. Three videos were sent during the didactic training sessions and then one video was then sent after each coaching session. All videos were recorded separately from the coaching sessions which allowed for a measure of parent’s ability to generalise skills to play sessions when the coach was not present.

#### Parent Dependent Variables

Parents were taught to incorporate three strategies into their play sessions. These were taught progressively (i.e., each strategy was added into the play sessions once mastery in the previous strategy was displayed). The main parental dependent variable guiding the progression of the study was procedural fidelity in the implementation of each strategy and data collected was used to answer the research question: “Can a telehealth model be used to train parents to use ABA based natural environment teaching strategies to a high level of fidelity?”. Fidelity checklists can be found in Table 3.

**Table 3:** Fidelity checklists and operational definitions for each strategy

Strategy step	Operational definition	Recording method
Strategy 1 step		
1. Play area is set up with the child’s favourite items	Play area should contain at least 5 potentially preferred items.	Partial
2. Play shaped by motivation	Parents should wait for the child to initiate the play with any item. Initiation can include reaching for toy, looking towards parent to gain more of something (bubbles, tickles etc.). This can be scored if motivation has continued over from previous intervals or if new discrete episodes of child initiation are observed. If above behaviour is the result of a First-Then contingency to gain access to a different preferred activity, it will not be scored as motivation.	Partial
3. Offers a choice (If applicable)	If the child is not engaging with any toys, preferred items can be placed in front of the child or offered, by demonstrating the fun properties of the item.	Partial
4. Position is facing the child	Parents should position themselves facing the child unless the play does not allow for this, e.g. spinning etc.	Whole
5. Joined in play appropriately	Play should be joined in by adding on preferred items, no demands and demonstrating fun ways to play with items. Parents should not take over the play but should aim to follow their children’s lead. Prompting for eye gaze or mands are not considered demand.	Whole
6. Used language appropriately	Language should not involve demands and should be clear, concise and simple.	Whole
7. Reinforces desirable behaviour	Any desirable behaviours such as vocalisations, eye gaze, imitation should be reinforced by praise (e.g. nice looking, Wow you copied!) and the item or activity if appropriate or a natural continuation of the activity that would be reinforcing in itself (e.g. child looks at Mum and she nods her head, smiles and presents a fun play action).	Partial
Strategy 2 step		
1.Uses a motivation creation strategy	Uses a motivation creation such as: withholding items, providing small amount of item, adding on items to play. (Full descriptions can be found in Table 5).	Partial
2.Utilises child initiation	Child demonstrating an interest in the toy or activity, to which access is currently controlled by the parent. Initiation can include reaching for toy, looking towards parent to gain more of something (bubbles, tickles etc.), pulling a parent’s hand towards an item or activity or vocalising a sound, word approximation or whole word requests. Parents should wait for this initiation before prompting. Score N/A if child is not motivated to gain access to anything under the control of the parent and negative if prompting takes place without this initiation or if this initiation is present but not utilised by creating a teaching moment.	Partial
3. Uses correct prompt technique (If applicable)	Uses prompting as described in the strategy information sheet at correct prompt level. Prompts can include full sweep/ search. Partial sweep/ search and time delay.	Partial
4. Reinforces eye gaze	Provide access to the item or a natural continuation of the activity and additionally praise. Items used to contrive motivation should not be provided unless eye gaze has been observed or 3 unsuccessful attempts to prompt have been made. If reinforcer is provided after unsuccessful attempts, this should be of lesser magnitude or amount than if behaviour occurred.	Partial
Strategy 3 Step		
1.Uses a motivation creation strategy	As above.	Partial
2.Utilises child initiation	As above.	Partial
3. Eye gaze	Parents should gain eye gaze before prompting or providing access to item if mand was independent but eye gaze was not present.	Partial
3.Uses correct prompt technique (If applicable)	Uses prompting as described in the strategy information sheet (available on request). Prompts can include full echoic prompt, 3 and 5 s time delay.	Partial
4. Reinforces Communication	Reinforcement should include praise (e.g., nice speaking, beautiful words!) and the item or activity if appropriate or a natural continuation of the activity. Items used to contrive motivation should not be provided unless communication has been observed or 3 unsuccessful attempts to prompt have been made. If item is provided after unsuccessful attempts, this should be of lesser magnitude or amount than if behaviour occurred.	Partial

During Strategy 1 parents were taught to gauge and utilise the motivation of their child and use this to guide their play session. Additionally, they were taught to use simple language and minimise demand throughout. In Strategy 2 parents were trained to create motivation for items or continuation of play via environmental arrangements to teach eye gaze “mands”. The environmental arrangements can be found in Table 4. In Strategy 3 parents were taught to increase child manding using individualised communication targets. Again, parents had to create and utilise motivation using a strategy from Table 4 but were now taught how to add on to this by prompting a mand. Fidelity checklists were designed to assess parental performance throughout the whole session for each of the three strategies. Recording details and operational definitions for each checklist can be found in Table 3. Data were collected for each variable listed on the fidelity checklists every 20 s via interval recording; this ensured repeated measures were collected throughout the entire session. Additionally, checklists for Strategies 2 and 3 were designed to allow scoring of intervals during which opportunities for manding were not created (i.e., parents had to create a minimum of 12 attempted opportunities for communication across the session in order to achieve a mastery score) or when parents reacted correctly to child self-initiations towards preferred items which were not a result of the use of parents creating the motivation (e.g., if a child spontaneously requested “tickle” and the parent responded accordingly, they would be scored as correct for providing reinforcement but would score not applicable for creating motivation or prompting language as this was not required during this interval). These ensured enough opportunities were created within the sessions and parents were credited for reacting to their child’s motivation accordingly. Parents were introduced to each of the strategies in turn across the progression of the study, with the next strategy being introduced once parents displayed mastery of the previous strategy (i.e., two consecutive videos being scored at 80% or higher) (Table 5).

**Table 4:** Mean and range of parent and child IOA scores

Parent	Elaine	Christina	Jill	Diane	Sarah
Strategy 1% *M*	97	97	95	97	96
*Range*	96–99	96–98	90–98	94–98	92–99
Strategy 2% *M*	94	92	96	92	95
*Range*	91–95	90–93	90–100	90–96	93–97
Strategy 3% *M*	95	93	96	92	94
*Range*	92–96	91–94	95–97	86–99	90–96
Child	Patrick	Kostas	Leanne	Sean	Eamonn
Eye Gaze % *M*	94	94	96	89	98
*Range*	84–100	86–100	91–100	80–95	92–100
Communication % *M*	96	95	98	96	98
*Range*	86–100	89–97	90–100	91–100	94–100
Affect *M*	97	96	97	96	97
*Range*	90–100	90–100	93–100	93–100	96–98

**Table 5:** Descriptions and examples of each motivation creation strategy

Motivation Creation Strategy	Description	Examples
Add-on	Parents provide their child with objects or activities which can enhance their current play. They showed the objects in your hand or did something exciting with them and waited for their child to indicate that they would want them.	Playing with blocks, you can provide additional blocks needed to play by taking them out of a bag one by one and holding them out for your child. In a colouring activity you could provide additional crayons needed to colour in the picture.
Small amounts	Parents will provide a very small amount of the object or activity and wait for an indication they want more.	Whilst playing with Playdoh, you can give your child a small piece to play with, in order to created motivation for them to want more. During bubbles, blow a few bubbles only.
Pause-play	In this strategy parents start to play with their child and then suddenly stop and pause the play until they look and request. This works best with social games and activities, such as tickles, singing songs or blowing bubbles.	Providing tickles and suddenly stopping the game before starting again when eye gaze and request is provided. Dancing to a favourite song and pausing the music to wait for eye gaze and request.
Withhold items	Here you will deliberately withhold access to certain items that you know are needed to either finish a task or to play with the item.	Playing with a ball popper game, you start the game without the balls and wait for eye gaze and requests before providing access to the balls. Whilst completing a puzzle, you hold on to the last piece of the puzzle, or you keep hold of the crayons needed to colour a picture.
Need help	The environment is contrived in a way that often help is needed to gain access to preferred items. This help can be provided for locating an item that is out of reach of the child or for accessing an item that is kept items in a clear jar or ziplock bag.	Your child is really motivated to play with a ball, when you enter the playroom, the ball is located up high on a shelf out of reach of the child. You wait for your child to look at you and request before providing access. You are playing car ramps with your child, and you place the cars for the game in a see-through container that your child cannot open. Open the container when you get eye gaze and request.

The total number of correctly implemented steps was divided by the sum of correctly and incorrectly implemented steps to provide an overall percentage fidelity score for each strategy in each video including baseline, didactic probes, intervention and maintenance. Fidelity scores were graphed and visually analysed for changes in level, trend and variability. In addition to this effect sizes were calculated using Tau-U, a statistical model capable of determining effect size in single subject research by examining the percentage of non-overlap in data between conditions, whilst taking into consideration level and controlling for positive baseline trends (Parker et al., 2011). Estimated effect sizes can be calculated even when data points are minimal and can be rated as weak (< 0.66), medium (0.66–0.92) and large (> 0.92) (Parker & Vannest, 2009; Rispoli et al., 2013).

#### Child Dependent Variables

Occurrence of prompted and independent eye gaze was counted across the full 10-minute duration of each video to provide a total frequency score. Child eye gaze definition was based on prior research teaching this skill (e.g., Carbone et al., 2013) and was defined as “movement of the head and eyes that led to perceived contact with the eyes of the parent”. Prompted eye gaze included any attempt made by the parent which successfully got their child to look at them, including techniques provided in training such as sweeping and searching or techniques which were not trained but were present in the parent’s repertoire, although not encouraged once training began, such as moving the child’s head or saying, “look at me”.

Occurrence of an individualised manding target was the second child dependent variable. This was defined as “any attempt to request for an item or activity, for which access was being controlled by the parent and motivation was present in the child to obtain”. An overview of each child’s target is provided in Table 2. Patrick and Leanne’s targets were one-word vocal requests. As Sean had no prior vocalisations, his targets consisted in one-word signs (e.g., sign for ball); if any vocal requests occurred at any point during the training, these were immediately reinforced and counted as correct responses. For two participants, Kostas and Eamonn, targets included two to three-word mands. However, for both participants one-word requests were still reinforced and counted as correct responses. Parents would honour the request but build upon the language when prompting and modelling longer sentences straight after the initial mand was honoured and differentially reinforcing any two to three-word mands. Data collected on eye gaze and individualised communication targets were used to answer the question: “Does this training result in increased communication skills for the children?”

Occurrences of child positive affect were scored using 20 s partial interval recording. Positive affect was defined in behavioural terms and included behavioural indicators of enjoyment using a similar coding system to the one described by (Greiger et al., 2012); positive affect included “at least one instance of smiling (upward curve of the mouth, with or without showing teeth), making positive statements regarding the activity or laughing/giggling during the interval, not to coincide in time with any occurrences of behaviour that challenges”. If behaviour considered challenging was present within the same interval as the positive affect, the positive affect could still be counted as long as it did not occur alongside or as a result of said behaviour (e.g., a child giggling whilst pinching a parent would not be counted). Data collected on positive affect were used to answer the research question: “Does this training result in increased levels of positive affect for the children?”.

#### Interobserver Agreement (IOA)

A second rater scored at least 33% of videos for all dependent variables and experimental conditions; the rater was blind to which phase of the intervention the video corresponded to. The rater scored the pre-recorded videos submitted by the parents for all dependent variables. Prior to collecting IOA data for any participant the rater took part in training with the first author to discuss and practise the definitions of and the scoring criteria for each variable with each participant. This included identifying examples and non-examples of target behaviour as per past research measuring similar dependent variables via video recording (Tanner & Dounavi, 2020). Due to the large number of variables being collected at once, the rater would watch each video several times in order to score each variable consecutively and accurately. Scores of parental fidelity and child affect utilised interval by interval occurrence IOA, where the number of intervals in agreement over the presence of the behaviour, were compared with the overall number of intervals, resulting in a percentage agreement. The main child variables (eye gaze and mands) utilised total count IOA, where the smallest count was divided by the biggest count and multiplied by 100 to provide an agreement level for each variable in the session (Cooper et al., 2007). IOA was 95% (range: 86–100%) for parental fidelity scores, 97% (range: 90–100%) for child affect and 94% for eye gaze (range: 80–100%) and 97% for manding (range: 86–100%). Table 4 displays the average scores of each participants IOA for each variable.

#### Social Validity

**Fig. 1:**
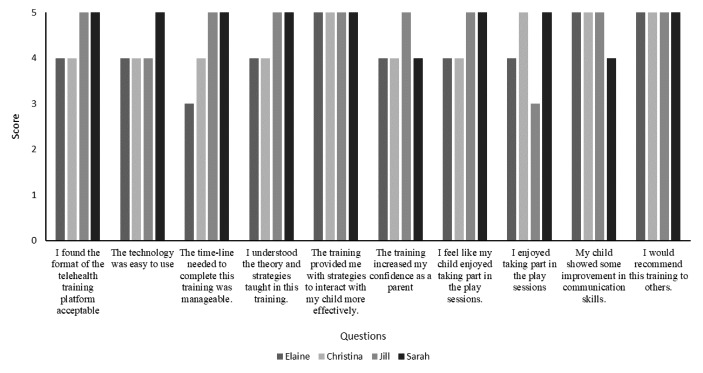
Social validity scores and questions

Quantitative and qualitative measures of social validity were collected upon the completion of the study. This included a questionnaire and an interview measuring parental views of the intervention. The questionnaire was adapted from the behaviour intervention rating scale (Elliott & Treuting, 1991) using a similar formatting for the questions and the use of a 5-point Likert scale (1 = strongly disagree, 2 = disagree, 3 = neutral, 4 = agree and 5 = strongly agree), but was individualised to include questions regarding the strategies taught, use of technology and child progress. Specific questions can be found in Fig. 1. Parents were also invited to provide written feedback if they so wished.

#### Cost Analysis

The cost analysis followed a similar methodology to the one employed by Lindgren and colleagues (2016), but did not include a statistical analysis of the different costs associated with each delivery method (e.g., telehealth vs. face-to-face). Costs of completing the parent training program were calculated for the telehealth model and compared with a hypothesised face-to-face model. For the telehealth model this included: (1) cost of purchasing and shipping the hardware, (2) cost of BCBA® direct “in session time”, which were calculate as the time for each video conferencing meeting and (3) cost of BCBA® indirect data collection time, calculated at 0.5 h per video submitted. An estimation of the cost of conducting the project in a face-to-face manner was also calculated and included: (1) cost of travel (one visit per each coaching session plus one visit for assessment and one for each didactic training session), (2) indirect cost of time BCBA® would spend travelling, and (3) cost of BCBA® direct “in session time”. As data collection could be conducted in session during a face-to-face model, this time was not included in the estimate. Costs of BCBA® hourly rate were taken from appropriate literature (Dounavi et al., 2019) and travel times and distances were calculated using Google Maps © software. The cost of milage was calculated using the cost of 0.45 pence per mile, which is the cost provided by the UK government (UK Government, 2021).

### Procedures

#### Didactic Training

The initial training phase involved six 1-hour long didactic training sessions designed to provide an overview of the theory of ABA. For each session parents took part in an hour-long synchronous session with the trainer delivered via Skype™. During this meeting parents were talked through a presentation delivered on Prezi© software. Each presentation started with a recap of the previous session and ended with a short, spoken quiz. In addition to the presentation, for each session parents were provided with a PDF document outlining the topics discussed in more detail. After completing each session and prior to the next session, parents were asked to complete one activity (e.g., a matching activity, fill in the blanks or completion of the Child Preference Inventory) and one 10-question multiple-choice test. Parents were free to work their way through this independently. In order to progress to the next session parents were required to score at least 80% in the multiple-choice test. If they scored less than this a recap was provided at the start of the next session and they were asked to sit the test again. Subsequent sessions were locked until this score was achieved. This only occurred for one parent on one session. At the end of the didactic training parents were asked to re-sit the multiple-choice test in order to proceed to the live coaching sessions and a scores of 80% or higher was needed, this was achieved by all parents on the first attempt. An overview of the topics covered in the training can be found in the supplementary material (Supplementary Table 3).

#### Live Coaching

Coaching sessions took place at least once per week and twice per week when the parents’ scheduling allowed. Prior to starting the coaching sessions, parents were provided with written instructions on how to set up the play area, including how to incorporate preferred items in the area. Parents were coached to include the three strategies in their play sessions: (1) gauging and building upon child motivation with simple language, (2) creating motivation to teach eye gaze, and (3) creating motivation to teach individualised mands. Coaching in each strategy closely followed the fidelity checklists (Table 3). Positive feedback was provided when parents exhibited behaviour which closely reflected the fidelity checklist and suggestions were made to error correct any behaviour which differed. Each strategy was introduced progressively into the play sessions until parents demonstrated the mastery criterion of the strategy in their videos (two videos at ≥ 80%). After this time the next strategy was added into the play session, however due to the strategies not being functionally distinct from each other (e.g., teaching parents to face their child may also help them to teach eye gaze or creating motivation to teach eye gaze and mands used the same strategies) and due to parents continuing to include the previously mastered strategies in their play, parents received ongoing coaching which continued to include previously mastered strategies throughout.

The first coaching session of each new strategy involved two training components. The first component was a didactic overview of the procedure, including a PDF written rationale, flow diagram, checklist including all the expected behaviours and a video example demonstrating the use of the strategy. In the second component, parents were coached to practise the strategies with their child. Subsequent coaching sessions involved spoken feedback of the most recent video submission and further coached practice. Sessions aimed to be 1-hour long, including time for feedback at the start and end, however the average length of the coaching sessions was 48 min. Coaching sessions continued until parents had demonstrated mastery in all three strategies. The average total time taken for parents to complete the main body of the project, including didactic training and coaching was 3.7 months (Range: 104–123 days).

#### Maintenance

After the completion of the project, parents were asked to send follow-up video probes using the target strategies. Probes were collected monthly for 3 months, although delays in receiving videos resulted in the maintenance period lasting longer than the planned 3 months for the first two participants. Jill sent one probe and Sarah sent two follow-up probes. Although, the majority of the study was completed in 2019 prior to the outbreak of the Covid-19 pandemic, follow up probes for the final two participants were collected after this time. Due to this, Diane had a change in life circumstances following the completion of the project and was unable to send videos or take part in feedback meetings due to lack of internet access.

## Results

### Parent Dependent Variables

**Fig. 2:**
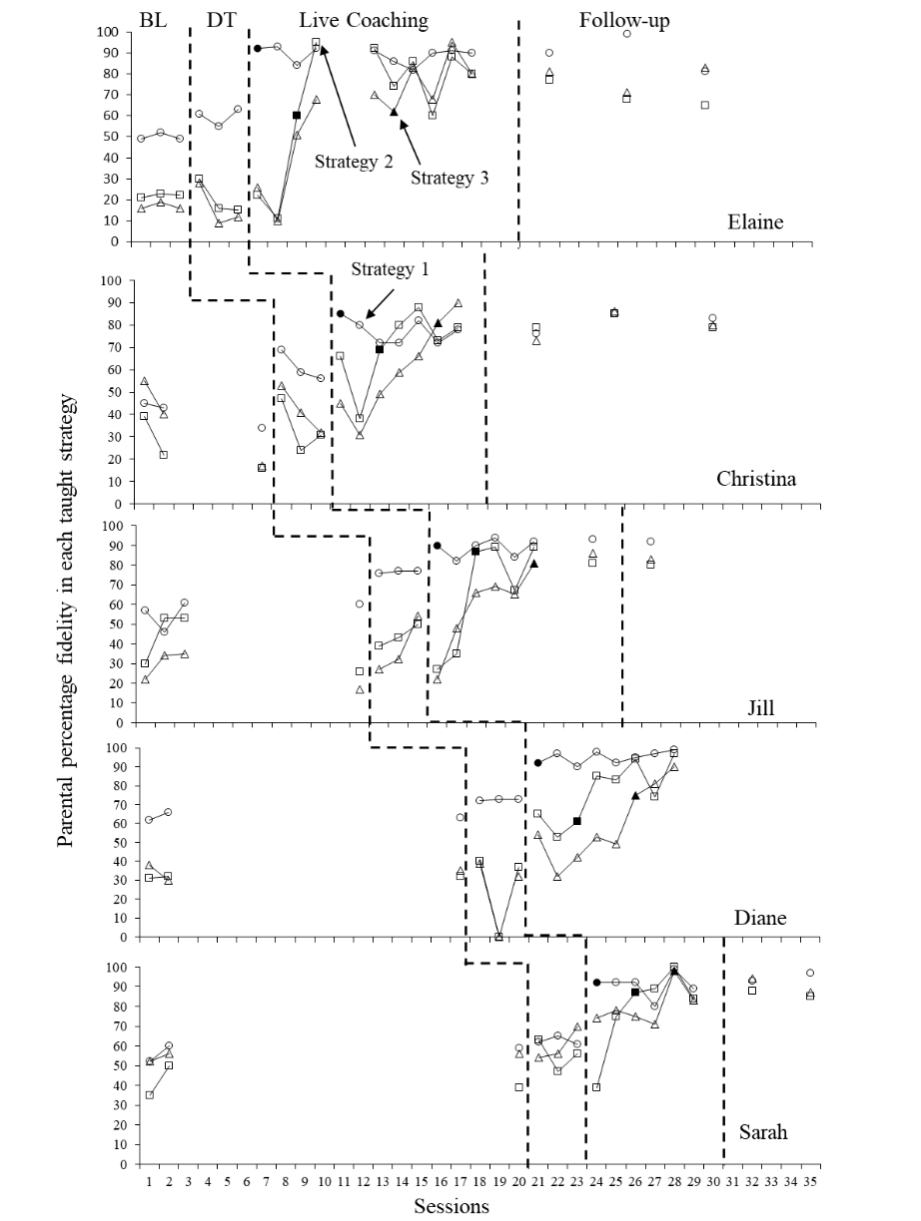
Parental fidelity in implementing each taught strategy. *Legend*: BL = Baseline DT = Didactic training Filled shapes indicate scores for first video sent after training and coaching in the strategy was introduced. Gaps in data for Elaine and Jill represent time away from the project

Parental fidelity in implementing all three strategies across baseline, didactic probes, intervention and maintenance is displayed in Fig. 2. Visual analysis for all participants suggests that there was a functional relation between the training and observed increases in parental fidelity. As each strategy is not functionally distinct, the relation between the introduction of each coaching strategy will be examined in turn with respect to all three strategy fidelity scores.

### Fidelity Scores Across Strategies

During coaching in the first strategy, parents were taught to gauge and utilise the motivation of their child and to allow this to guide the progression of the play session, whilst adding simple language and minimising demand throughout. Individual and combined fidelity score means and ranges for all strategies can be found in supplementary material (Supplementary Table 4). The introduction of coaching sessions resulted in immediate increases in all parents’ scores, with every parent now exhibiting a score ≥ 80% directly after the first coaching session. All parents met the mastery criterion within two sessions. This change was maintained across all subsequent coaching sessions and introductions of subsequent training strategies for all parents apart from Christina whose scores fell just below 80% during her last two sessions. Individual Tau-U scores from baseline to after coaching has commenced indicated strong effect sizes for all participants (Elaine: Tau-U = 1, z = 2.54, p = 0.01 with 90% CI [0.35,1]; Christina: Tau-U = 1, z = 2.39, p = 0.02 with 90% CI [0.31,1]; Jill: Tau-U = 1, z = 2.65, p < 0.01 with 90% CI [0.35,1]; Diane: Tau-U = 1, z = 2.45, p = 0.01 with 90% CI [0.33,1] and Sarah: Tau-U = 1, z = 2.32, p = 0.02 with 90% CI [0.29,1]). The weighted Tau-U effect size suggests a statistically significant strong effect size for combined parental scores: Tau-U = 1, z = 5.51, p < 0.001 with 95% CI [0.64,1].

Parents were now taught Strategy 2; to create motivation for items or continuation of play via environmental arrangements to encourage eye gaze “mands”. None of the parents scored ≥ 80% during any of the didactic probes for Strategy 2. Upon the commencement of the coaching phase parents were first taught to include Strategy 1 into their play. Although this did not directly involve steps from the fidelity checklist of Strategy 2, there were some increases evident upon visual analysis of the data as some of the steps in this strategy may facilitate opportunities for eye gaze to be included in play (e.g., sitting opposite the child or providing the child with extra toys to add to the play). Strategy 2 specific coaching was then introduced. Visual analysis of data indicated that this resulted in an immediate increase in level for all participants apart from Diane, who required one more session before an increase in level was apparent. Once again, individual Tau-U scores from baseline to after coaching has commenced indicated a strong effect size for all participants (Elaine: Tau-U = 1, z = 2.45, p = 0.01 with 90% CI [0.33,1]; Christina: Tau-U = 1, z = 2.23, p = 0.03 with 90% CI [0.24,1]; Jill: Tau-U = 1, z = 2.45, p = 0.01 with 90% CI [0.33,1]; Diane: Tau-U = 1, z = 2.32, p = 0.02 with 90% CI [0.29,1] and Sarah: Tau-U = 1, z = 2.12, p = 0.03 with 90% CI [0.23,1]). The weighted Tau-U estimates a statistically significant strong effect size for combined parental scores: Tau-U = 1, z = 5.16, *p* < 0.001 with 95% CI [0.62,1]. Alongside visual analysis of each parent’s data this indicated a strong functional relation between coaching and increased fidelity.

 During Strategy 3 parents were taught to increase individualised mands. Three parents demonstrated an increase level in their scores and all but Sarah demonstrated a change from a stable to an increasing trend. When parents were then introduced to coaching directly in the implementation of Strategy 3 all reached ≥ 80% within the first two training sessions. Individual Tau-U scores from baseline to after coaching in Strategy 3 started, once again indicated a strong effect size for all participants, although the smaller number of data points for this measure limited the capabilities of the test to determine statistically significant findings at high levels of confidence for Christina, Jill and Sarah (Elaine: Tau-U = 1, z = 2.23, p = 0.03 with 90% CI [0.26,1]; Christina: Tau-U = 1, z = 1.73, p = 0.08 with 90% CI [0.05,1]; Jill: Tau-U = 1, z = 1.85, p = 0.06 with 90% CI [0.11,1]; Diane: Tau-U = 1, z = 1.96, p = 0.04 with 90% CI [0.16,1] and Sarah: Tau-U = 1, z = 1.73, p = 0.08 with 90% CI [0.05,1]). The weighted Tau-U from baseline to after coaching in strategy 3 commenced suggests a statistically significant strong effect size for combined parental scores: Tau-U = 1, z = 4.20, *p* < 0.001 with 95% CI [0.53,1]. This supports the visual analysis in confirming that there was a functional relation between coaching in this strategy and increased levels of parent fidelity.

#### Follow-up Probes

 All parents who sent follow-up probes maintained ≥ 80% fidelity on the first strategy on all follow-up probes apart from Christina whose score dropped to 79% on the first probe. Christina, Jill and Sarah maintained levels above 80% for all probes of using Strategy 2, whereas Elaine dropped below 80% during the final two probes. Jill and Sarah’s scores in Strategy 3 remained above 80%, Elaine’s dropped to 68% during her second probe but returned to 81% in her third probe. Christina’s first probe was 73% but her remaining two probes were ≥ 80%. It is important to note though that all scores remained far above baseline levels for all participants in all strategies.

### Child Dependent Variables

Child dependent variables were not used in decision making regarding progression of the study. Both primary and distal child dependent variables were graphed and analysed for functional relations by looking at trend, level, variability and immediacy of effect. Means and ranges for eye gaze and mands are displayed in the supplementary materials (Supplementary Table 5). Additionally, overall effect sizes were calculated using Tau-U.

#### Eye Gaze

**Fig. 3:**
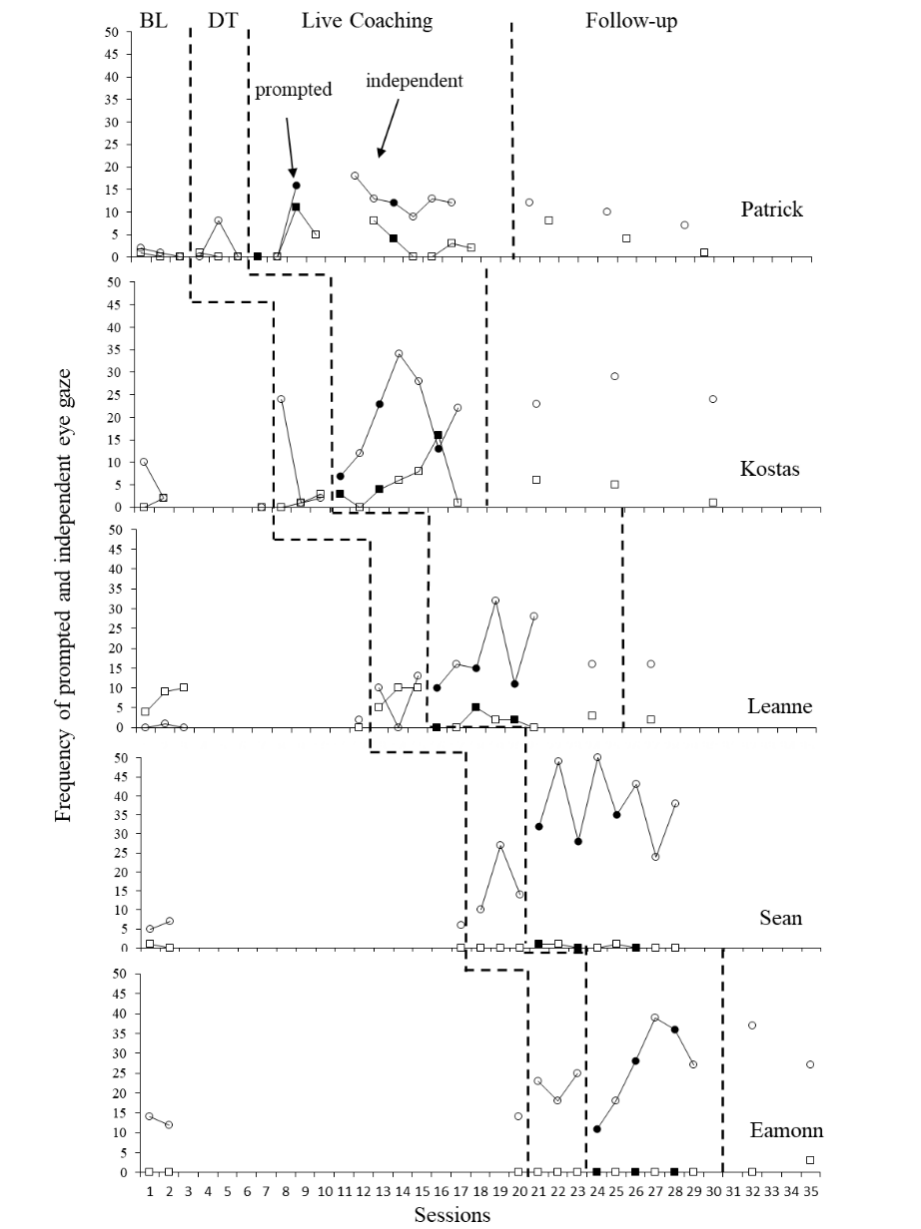
Frequency of prompted and independent child eye gaze per 10-minute video. *Legend*: BL = Baseline DT = Didactic training. Gaps in data for Patrick and Leanne represent time away from the project. Filled shapes indicate sessions directly after a parent was introduced to a new coaching session

Prompted and independent occurrences of eye gaze were scored across each recorded play session and can be seen in Fig. 3. Baseline levels of independent eye gaze were relatively low for all child participants and were stable for all participants apart from Kostas, who demonstrated a counter therapeutic trend in independent eye gaze but a stable trend in prompted eye gaze. All participants saw some increase in independent eye gaze at some point during the didactic probes. When parents were introduced Strategy 2, focusing on how to teach eye gaze, all children displayed an increase in levels of independent eye gaze within the first two coaching sessions. Patrick displayed an initial increase in prompted eye gaze, which was followed by a subsequent increase in independent eye gaze after the following session. Levels remained elevated for the remainder of the intervention for all participants, including during the follow-up videos. Visual inspection of the combined data across the group indicates a functional relation between training provided to parents and observed increases in eye gaze. This relation was further supported by large effect size estimates in independent eye gaze for all children apart from Patrick (Patrick: Tau-U = 0.75, z = 1.83, p = 0.06 with 90% CI [0.078,1]; Kostas: Tau-U = 1, z = 2.23, p = 0.03 with 90% CI [0.26,1]; Leanne: Tau-U = 1, z = 2.44, p = 0.01 with 90% CI [0.33,1]; Sean: Tau-U = 1, z = 3.32, p = 0.02 with 90% CI [0.29,1]; and Eamonn: Tau-U = 1, z = 2.12, p = 0.03 with 90% CI [0.22,1]). Patrick’s increases in total eye gaze did indicate a strong effect size (Tau-U = 1, z = 2.45, p = 0.01 with 90% CI [0.33,1]). When all scores on independent eye gaze were combined, an estimated strong effect size was observed between baseline and post coaching scores (Tau-U = 0.95, z = 4.88, *p* < 0.001 with 95% CI [0.57,1]).

#### Child Communication

**Fig. 4:**
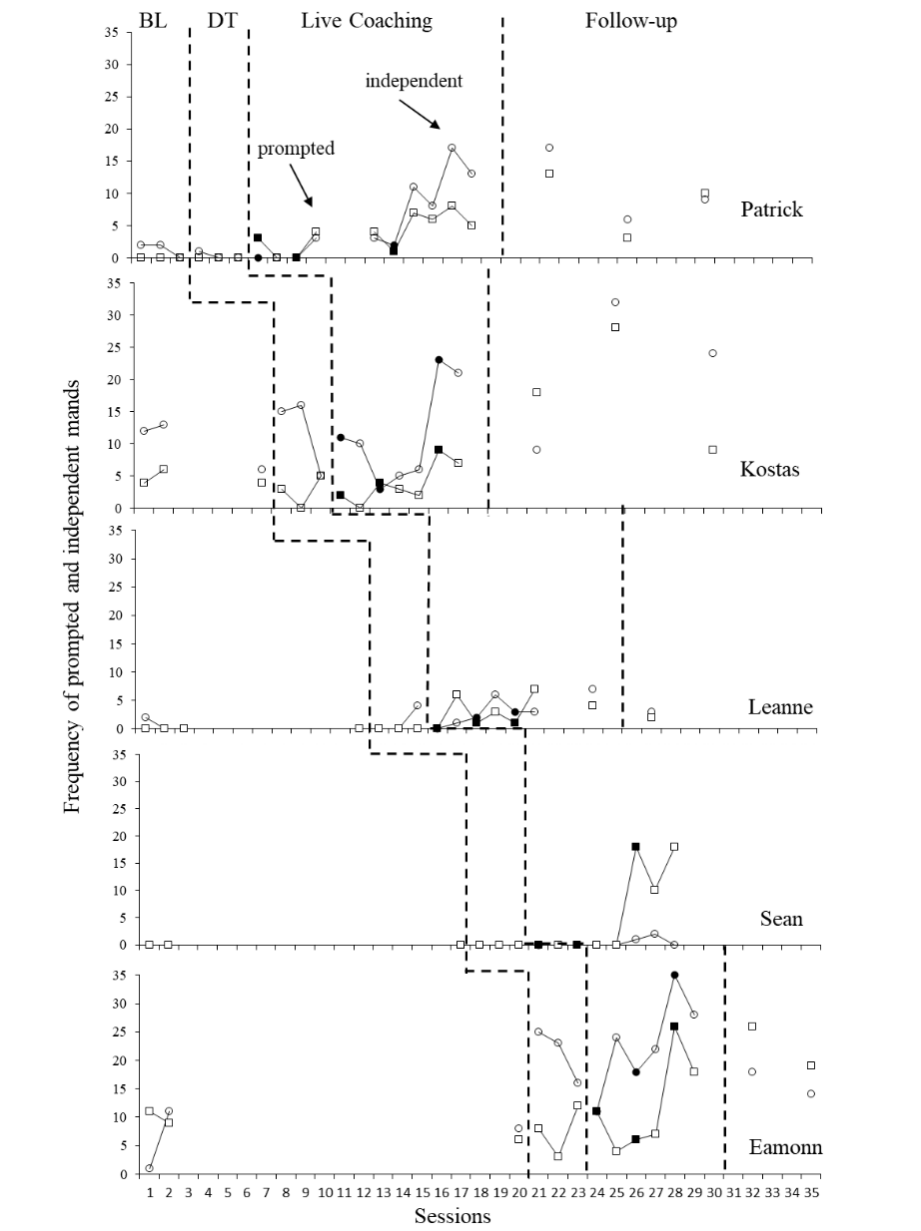
Frequency of prompted and independent child mands per 10-minute video. *Legend*: BL = Baseline DT = Didactic training. Gaps in data for Patrick and Leanne represent time away from the project. Filled shapes indicate sessions directly after a parent was introduced to a new coaching session

Prompted and independent occurrences of child manding were scored (see Table 2 for individualised targets and Fig. 4 for data). Baseline levels of mands varied somewhat between children. When parents were introduced to didactic training, Eamonn was the only child to display an increased level in manding. When parents were coached to increase eye gaze Patrick and Leanne demonstrated a small increase in vocal mand frequency. Each parent was then coached how to add Strategy 3 into their play sessions, for all children this resulted in an increase in either independent or prompted mands within the first two sessions. At follow-up only Kostas’ independent mands remained at levels seen in the final coaching sessions. Both Patrick and Eamonn displayed mands at a level which was higher than during baseline but was reduced slightly from the final coaching sessions. Leanne’s level had dropped back down to be only slightly above her baseline level.

Visual inspection of data across participants suggests that the introduction of the intervention resulted in an increase in the level of overall manding for all participants when compared with their baseline scores. This indicates that there was a functional relation between coaching and observed increases in overall manding. As it was parental data rather than child data guiding the progression of the intervention and due to a larger amount of variability in the data, this conclusion cannot be as confidently reached as with parental results. However, large individual effect sizes were estimated for all increases in total mands (Patrick: Tau-U = 1, z = 2.24, p = 0.03 with 90% CI [0.26,1]; Kostas: Tau-U = 1, z = 1.73, p = 0.08 with 90% CI [0.05,1]; Leanne: Tau-U = 1, z = 2.12, p = 0.04 with 90% CI [0.23,1]; Sean: Tau-U = 1, z = 1.96, p = 0.05 with 90% CI [0.16,1]; and Eamonn: Tau-U = 1, z = 1.73, p = 0.08 with 90% CI [0.050,1]). For Kostas and Eamonn increases in total mands were not considered statistically significant due to having fewer data points available during the coaching phase. Scores for combined totals mands also indicated a large effect size (Tau-U = 1, z = 4.31, *p* < 0.001 with 95% CI [0.55,1]).

#### Positive Affect

**Fig. 5:**
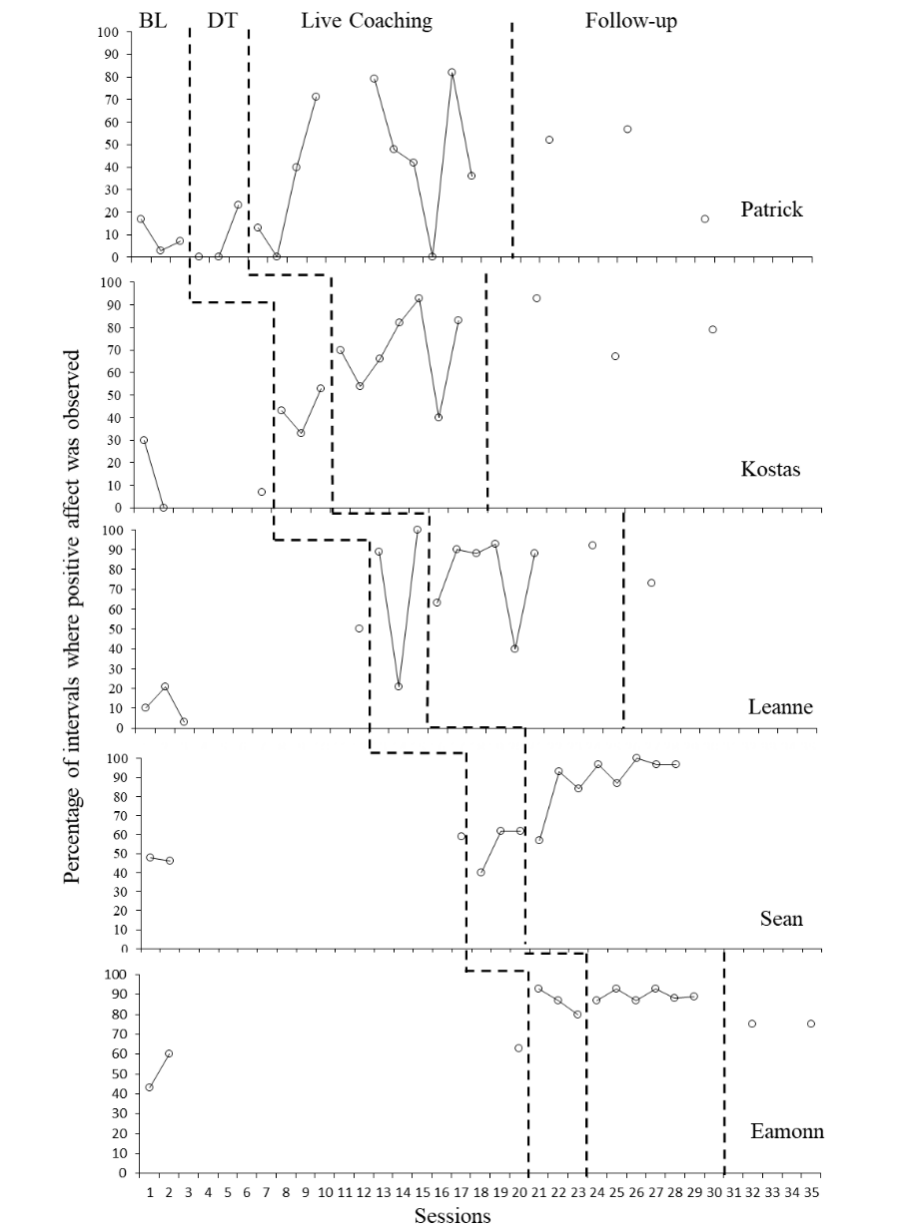
Percentage of intervals where child positive affect was observed. *Legend*: BL = Baseline DT = Didactic training. Gaps in data for Patrick and Leanne represent time away from the project

Figure 5 shows the percentage of intervals during which the child displayed positive affect throughout the progression of the study. Visual inspection of data across participants suggests that the introduction of either the didactic training or coaching resulted in an increase in the level of positive affect for all participants when compared with baseline scores. The use of statistical analysis that corrected any positive baseline trends increased our confidence in this observation. Individual Tau-U effect size calculations indicated a strong effect size for Kostas, a medium effect size for Leanne, Sean and Eamonn and a small effect size for Patrick (Patrick: Tau-U = 1, z = 1.35, p = 0.18 with 90% CI [-0.12,1]; Kostas: Tau-U = 1, z = 2.39, p = 0.02 with 90% CI [0.31,1]; Leanne: Tau-U = 0.86, z = 2.27, p = 0.02 with 90% CI [0.24,1]; Sean: Tau-U = 0.88, z = 2.14, p = 0.03 with 90% CI [0.20,1]; and Eamonn: Tau-U = 0.83, z = 1.94, p = 0.05 with 90% CI [0.125,1]). When the combined data were analysed, a medium effect size became apparent (Tau-U = 0.82, z = 4.5, *p* < 0.001 with 95% CI [0.46,1]).

### Social Validity

 Four out of five parents filled in a 10-question 5-point Likert scale questionnaire, designed to measure the acceptability of the training materials, delivery platform, strategies and meaningful changes. Results suggest high acceptability of the intervention, materials and platform, as well as satisfaction with achieved behaviour change (Fig. 1). Data indicated that parents were very pleased with the study’s procedures, responding “agree” or “strongly agree” to all questions but two. Elaine scored “not sure” for the question relating to the acceptability of the timescale and Jill rated “not sure” to whether she found the training enjoyable. Both parents clarified the score with longer written answers or during the interview. Elaine stated that she felt some unexpected life experiences got in the way of her ability to complete the project on the planned timeline and Jill stated that she found it quite upsetting to watch her daughter on video and witness her diagnosis in this way.

### Cost Analysis

Table 6 presents a cost savings analysis for running the project via telehealth versus a face-to-face model. When travel costs and BCBA® costs of time spent travelling were taken into consideration, there were considerable savings when the project was run via telehealth. This totalled £7,546.7 across the duration of the project, which was an average of £1,509.3 per parent.

**Table 6:** Cost analysis comparing cost of telehealth and face-face model

Face-to-Face					Telehealth			
Participant	Round Trip Distance/ time	Cost of mileage/ flight per session	Cost for travel across project	Cost BCBA time (direct hours + travel time x average hourly cost- £45*)	Equipment cost (Including postage)	Cost BCBA time direct labour (hours x average hourly cost)	Cost BCBA time indirect labour (hours x average hourly cost)	Savings from face-face model across duration of the project
Elaine	55 miles 1 h 6 min	45 pence/ mile x 55 = £24.8	£24.8 × 12 = £297	(18 x £45) + (13.2 x £45) £810 + £594 = £1,404	£83	18 x £45 = £ 810	8 x £45= £360	£ 448
Christina	576 miles Approximately 4 h	Return flight = £ 100	£100 × 9 = £900	(15 x £45) + (36 x £45) £675 + £1,620 = £2,295	£85	15 x £45= £675	6.5 x £45 £292.5	£2142.5
Jill	276.2 miles 4 h 45 min	45 pence/ mile x 276.2 = £ 124.3	£124.3 × 9 = £1118.6	(15 x £45) + (42.75 x £45) £675 + £1,923.75 = £2,598.75	£85	15 x £45= £675	7 x £45= £315	£2639.4
Diane	77.4 miles 1 h 36 min	45 pence/ mile x 77.4 = £ 34.8	£ 34.8 × 11 = £ 382.8	(16 x £45) + (17.6 x £45) £720 + £792 = £1,593	£83	16 x £45 = £720	7 x £45 = £315	£557.6
Sarah	202 miles 4 h 12 min	45 pence/ mile x 202 = £ 90.9	£ 90.9 × 8 = £727.2	(14 x £45) + (33.6 x £45) £720 + £1512 = 2,232	N/A	14 x £45 = £630	6 x £45 = £270	£1759.2
								£7546.7

*Average cost of £45 BCBA/hour taken from (Dounavi, Fennell, & Early 2019) Exchange rate calculated July 2020 (€1 = £ 0.9)

## Discussion

The primary aim of the current study was to determine if an international telehealth training platform could be used to effectively train parents to implement naturalistic teaching strategies into their play. Gains observed in all parental fidelity scores across all strategies suggested a high efficacy of the training. These gains echoed the findings of prior research from US-based studies (e.g., Ingersoll et al., 2016; Vismara et al., 2009) and provided a unique example where both trainers and participants were located outside of the US. This demonstration highlights the potential of telehealth utilisation on a global scale and as a promising solution to global shortages in expertise, which will undoubtedly become a more pressing matter with the imminent changes to global certification. Results were also consistent with past research showing a coaching component is a vital addition to telehealth training (e.g., Craig et al., 2021; Ingersoll et al., 2016; Meadan et al., 2016) as although parents displayed some increases in fidelity following didactic training alone, these were not indicative of a clinically significant level of behaviour change. The real-life application of the project was an excellent fit with the telehealth training platform. Parents were able to directly receive training into environments in which learned strategies could be readily used and were able to demonstrate their use in recorded sessions without the BCBA® present. The training was focused on child motivation and parents were taught how to recognise, create and utilise motivation in each of their play sessions, using toys and resource readily available within their home. This ensured behaviour change was generalised into everyday scenarios throughout, an important component in bridging the gap between behaviour analytic research and real-life applications.

The secondary aims of the study were to assess the effects of this parent training model on child outcome measures in social communication (eye gaze and manding) and child affect, to answer the questions: “Does this training result in increased communication skills for the children?” and “Does this training result in increased levels of positive affect for the children?”. All children demonstrated an increase in independent eye gaze across the progression of the study. Eye gaze can be a valuable behaviour to target in early intervention, as it is an important factor in the early development of communication skills and may be a precursor to further development in this area (Tanner & Dounavi, 2020). Restrictions of eye contact in early childhood can lead to the loss of many crucial learning opportunities, including opportunities to learn social communication behaviour and the “rules” of conversation (Carbone et al., 2013; Krstovska-Guerrero & Jones, 2016). One proposed mechanism for this is that social interactions do not function as reinforcers in the early development of children diagnosed with ASD (Bottini, 2018; Chevallier et al., 2012). Teaching eye gaze as a form of manding could help to predicate the reinforcing qualities of such interaction, which could have prodigious benefits. With the current study we have provided a demonstration of simple strategies which can be effectively taught directly into home environment and incorporated into everyday lives, utilising child motivation and allowing for initial demonstrations of contingency to occur after a low response effort behaviour.

All children demonstrated increases in either prompted or independent mands. However, variance between children was evident, as was the ability to successfully move from prompted to independent mands. Greater demonstrations of experimental control for all participants may have been evident if prompted and independent data had been combined, but this would have weakened the ability of the study to determine meaningful behaviour change. Some degree of variance is common in past research telehealth based naturalistic parent training packages (e.g., Meadan & Daczewitz, 2015; Vismara et al., 2013) but may also be indicative of research into social communication interventions in general. One recent review states there is no single intervention identified as effective at improving the social communication skills for every child with ASD (Watkins et al., 2017). Clinical judgement must be adopted to make the best choices based upon the resources available. The present study dedicated two sessions to conduct pre-assessments and interviews with parents. Dedicating more time would provide a more detailed clinical picture, perhaps resulting in more consistent child outcomes. For example, Sean was the only participant to be taught to use signs. He displayed increases in his prompted signs but demonstrated minimal ability to use them independently. The inability to fade out the prompts could be either a limitation of the telehealth platform, a lack of pre-requisite skills or indicative of the time constraints of the project. Past research has indicated that a total of 267 trials are required for the first independent sign to reach criterion (Scattone & Billhofer, 2008). If recommended parental practice outside of the coaching and filmed sessions did not occur, it would be unlikely that this target would have been met. Conducting a more detailed pre-assessment would have provided a better indication not only of Sean’s current skills set but a holistic picture of the resources available to the family. Future research could conduct a greater number of pre-assessment sessions or could alternatively hold face-to-face meetings prior to commencing training via telehealth.

Our study showed improved child affect. Measures of positive affect have been used as behavioural indicators of private events of ‘happiness’ (Ramey et al., 2019). To date this variable has not been included in past telehealth research. The measurement of this dependent variable allowed us to surmise that the use of motivational strategies in the project were received favourable by the children, thus providing a previously immeasurable indicator of child social validity. To increase confidence in these findings future research could adopt an individualised measurement system, capable of measuring each participant’s unique indicator of happiness.

The additional aims of the study related to parent social validity and cost effectiveness of the study. Parents rated the study favourably and additional parental feedback could be used to guide future research or service provision. For example, Jill indicted she did not enjoy making the videos as she found it uncomfortable to observe her interactions with her daughter. Jill had received the diagnosis for Leanne just prior to starting the project, this is valuable insight as to the difficulties of using this platform as a first means of contact with parents who have newly received diagnosis. It may be preferable to use a hybrid strategy where in-home support may be provided in the first instance. The cost analysis indicated that a telehealth platform could result in considerable savings. Which, as is the case for many European countries with a lack of funding for services, could have huge benefits for families who are forced to fund out of pocket (Keenan et al., 2015).

There were certainly limitations to the current study, some of which were a result of the telehealth platform. Problems occasionally arose around parental technological literacy and capability in using the technology employed in the studies. To combat these difficulties, we utilised a visual task analysis to assist parents; however, further assistance was occasionally required. Future research may want to consider measuring parental computer literacy prior to training and providing appropriate training as required. There were occasions when the connection was poor during the coached sessions. This resulted in poor picture quality or a time lag and caused issues in the interpreting behaviour and the ability to provide feedback in a timely manner. However, solutions were available, backup systems of connection were used, such as changing to mobile data. Additionally switching off the trainer’s video or providing delayed feedback reduced the impact but there is a risk that such measures could detrimentally affect the internal validity of the study as consistency across sessions was compromised.

Although it was not easy to collect data on eye gaze via telehealth, the study provides a framework to assist in this. This was doable due to the collection of pre-recorded videos rather than data collected from live sessions. Parents could spend time ensuring the best position of shot was used or use a second person to record if available. Additionally, there was little degradation in picture quality as would have been present had data been collected from the live coached sessions. Despite these solutions limitations do exist in these measures and they should be interpreted with caution. There may have been times when not all instances of eye gaze were captured in the video, for example when the child’s face could not be seen fully in shot. This may have led to an under estimation of eye gaze in earlier videos as parents were less exposed to the expectations of the video submissions. The fact that parents recorded videos at home could also introduce a data selection bias, in the case that parents watched videos and selected to share only those showing best outcomes. Additionally, limitations should be noted around the use of the total count IOA method and the levels of IOA reached, which were lower than the manding targets. Occurrences of eye gaze were very frequent towards the later stages of the project, the IOA system may not ensure the same occurrences of behaviour were being recorded. For this reason, future research may want to adopt an exact count per interval methodology for child dependent variables, this would create a more rigorous checking system.

An additional limitation is the lack of maintained behaviour change evident at follow-up. There could be several reasons for this. As targets were flexible to the child’s changing motivation, there were no rigid targets to teach across all sessions. This meant that new play scenarios would involve an increased level of prompting with a consecutive reduction in independent vocal verbal behaviour, there was certainly evidence of over-prompting in the follow-up videos. This could be rectified by fine-tuning training to further emphasise the use of time delay to fade prompts. The project adopted the parent fidelity scores as the main dependent variable; this was primarily due to time constraints but may have resulted in less-than-ideal demonstrations of control for the child outcomes and a reduced timeframe to ensure optimal behaviour change. Future researchers may wish to prioritise child behaviour change as the main dependent variable.

Further methodological limitations should be noted with the use of a probe design, chosen due to the very applied nature of the study. The study did not conform to the highest level of the What Works Clearing House Standards 4.1 (What Works Clearning House, 2020) for single subject research design as it did not have at least five data points in each baseline or consecutive data points prior to implementation (Horner et al., 2005). However, the baseline measures would meet the “with reservations” standard of this assessment and would also provide a high quality rating for this indicator in the “Evaluative Method for Evaluating and Determining Evidence-Based Practices in Autism” (Reichow et al., 2008). Again, due to the real-life application of the research it may not have been pragmatic to collect such a high number of videos just prior to starting the training and before rapport had been built with each family. For this reason, probes were conducted instead of continuous measurements of dependent variables prior to the start of the intervention. Limitations around the cost analysis should also be taken into consideration. Costs for home-based visits were calculated as the BCBA® visiting one family per trip, although this may not be the case in real-life, where the BCBA® may visit several families at one time. As parents utilised their own internet these were not factored into the calculation, however if parents’ internet speeds were not sufficient the cost of upgrade should be considered.

In sum, the current study demonstrated the effective use of a well-received telehealth training platform to provide parent training in naturalistic behavioural strategies, showing a rare application of this model in a European setting. Although gains were made in child communication and affect, future research may focus on prioritising child outcomes as the main dependent variable guiding the progression of the study, allowing for a greater confidence in the effects of the intervention on child outcomes. A telehealth training platform should be considered by behaviour analysts to expand their knowledge and increase the reach of their services, which could have benefits for parents who are not able to avail of training locally. Additional cost savings identified highlight the potential of a telehealth platform for service provision.

## Declarations

The Department for the Economy (DfE) funded two Postgraduate Research Studentships which supported the completion of the present study in fulfilment of the first and third authors’ PhD and under the supervision of the second author.

This study has been granted ethical approval by the Ethics Committee of the University where the study was completed. All procedures performed in studies involving human participants were in accordance with the ethical standards of the institutional research ethics committee and with the 1964 Helsinki declaration and its later amendments or comparable ethical standards.

Informed consent was obtained by all participating parents.
